# Acute Axonal Degeneration Drives Development of Cognitive, Motor, and Visual Deficits after Blast-Mediated Traumatic Brain Injury in Mice

**DOI:** 10.1523/ENEURO.0220-16.2016

**Published:** 2016-10-31

**Authors:** Terry C. Yin, Jaymie R. Voorhees, Rachel M. Genova, Kevin C. Davis, Ashley M. Madison, Jeremiah K. Britt, Coral J. Cintrón-Pérez, Latisha McDaniel, Matthew M. Harper, Andrew A. Pieper

**Affiliations:** 1 Department of Psychiatry, University of Iowa Carver College of Medicine, Iowa City, IA 52242; 2 Department of Psychiatry, Interdisciplinary Graduate Program in Human Toxicology, University of Iowa Carver College of Medicine, Iowa City, IA 52242; 3 Department of Veteran Affairs Center for the Prevention and Treatment of Visual Loss, University of Iowa Carver College of Medicine, Iowa City, IA 52242; 4 Department of Ophthalmology and Visual Sciences, University of Iowa Carver College of Medicine, Iowa City, IA 52242; 5 Department of Neurology, University of Iowa Carver College of Medicine, Iowa City, IA 52242; 6 Department of Free Radical and Radiation Biology, University of Iowa Carver College of Medicine, Iowa City, IA 52242; 7 Department of Veteran Affairs, University of Iowa Carver College of Medicine, Iowa City, IA 52242; 8 Weill Cornell Autism Research Program, Weill Cornell Medicine, Cornell University, New York, NY 10065

**Keywords:** axonal degeneration, nicotinamide adenine dinucleotide, neurodegeneration, traumatic brain injury, WldS mouse

## Abstract

Axonal degeneration is a prominent feature of many forms of neurodegeneration, and also an early event in blast-mediated traumatic brain injury (TBI), the signature injury of soldiers in Iraq and Afghanistan. It is not known, however, whether this axonal degeneration is what drives development of subsequent neurologic deficits after the injury. The Wallerian degeneration slow strain (*WldS*) of mice is resistant to some forms of axonal degeneration because of a triplicated fusion gene encoding the first 70 amino acids of Ufd2a, a ubiquitin-chain assembly factor, that is linked to the complete coding sequence of nicotinamide mononucleotide adenylyltransferase 1 (NMAT1). Here, we demonstrate that resistance of *WldS* mice to axonal degeneration after blast-mediated TBI is associated with preserved function in hippocampal-dependent spatial memory, cerebellar-dependent motor balance, and retinal and optic nerve–dependent visual function. Thus, early axonal degeneration is likely a critical driver of subsequent neurobehavioral complications of blast-mediated TBI. Future therapeutic strategies targeted specifically at mitigating axonal degeneration may provide a uniquely beneficial approach to treating patients suffering from the effects of blast-mediated TBI.

## Significance Statement

Blast-mediated traumatic brain injury (TBI) is the signature injury of soldiers associated with chronic cognitive, motor, and visual dysfunction. An early event in blast-TBI is diffuse axonal damage, but it is not known whether this drives development of subsequent pathology. *WldS* mutant mice are resistant to axonal degeneration via a mutation that enables maintenance of neuronal nicotinamide adenine dinucleotide (NAD) levels after injury, and a number of different approaches have been proposed for augmenting NAD levels in the nervous system. We show that *WldS* mice are protected from axonal degeneration and deficits in cognition, movement, and vision after blast-TBI. Axonal degeneration is thus a critical early event in this prevalent injury, suggesting therapeutic potential of specifically mitigating early axonal degeneration after blast-TBI.

## Introduction

Traumatic brain injury (TBI) is a leading cause of death and disability worldwide (Fleminger and Ponsford, 2005), with blast-mediated injury being the most common cause of TBI sustained by soldiers in the recent wars in Iraq (Operation Iraqi Freedom) and Afghanistan (Operation Enduring Freedom). Blast-mediated TBI places patients at risk for both acute and long-term neurologic complications, such as cognitive dysfunction, motor decline, psychiatric conditions, visual deficits, and neuropathologic features similar to Alzheimer’s disease (Hoge et al., 2008; Wolf et al., 2009; Goldstein et al., 2012; Shively et al., 2012). Sadly, there are currently no treatment options for patients beyond supportive and rehabilitative care.

Sheer forces associated with blast injury lead to widespread, diffuse, and progressive axonal injury, known to play a role in multiple forms of neurodegeneration (Raff et al., 2002; Nakagawa et al., 2011; Lingor et al., 2012; Magnuson et al., 2012; Yin et al., 2014). This form of injury and its associated behavioral deficits can be recapitulated in rodent models, which may therefore be useful for discovery and validation of new therapeutic approaches (Goldstein et al., 2012; Mohan et al., 2013; Yin et al., 2014). Pharmcologic agents shown to enhance flux of the nicotinamide adenine dinucleotide (NAD) salvage pathway in normal mammalian cells and facilitate NAD rebound following doxorubicin exposure (Pieper et al., 2010, 2014; MacMillan et al, 2011; Wang et al., 2014) confer protective efficacy on pathology and behavior in a rodent model of blast-mediated TBI (Yin et al., 2014), as well as other models of neurodegeneration in the central and peripheral nervous systems (De Jesús-Cortés et al., 2012, 2015,2016; Tesla et al., 2012; Blaya et al., 2014; Dutca et al., 2014; Naidoo et al., 2014; Kemp et al., 2015; Walker et al., 2015; Lee et al., 2016). In addition, treatment with NAD and NAD precursors, including nicotinamide, nicotonic acid mononucleotide, and nicotinamide mononucleotide (NMN), or overexpression of nicotinamide phosphoribosyltransferase protect axons *in vitro* (Araki et al., 2004; Wang et al., 2005; Sasaki et al., 2006).

To investigate whether NAD metabolism might be related to blast-mediated axonal degeneration in the brain, we applied the blast model of TBI to the Wallerian degeneration slow strain (*WldS*) of mice (Lunn et al., 1989). These mice were originally identified as being resistant to axonal degeneration after injury, and the *wlds* gene was subsequently shown to exist as a triplicated fusion gene encoding the first 70 amino acids of Ufd2a, a ubiquitin-chain assembly factor, that is linked directly to the complete coding sequence of nicotinamide mononucleotide adenylyl transferase 1 (Coleman et al., 1998; Conforti et al., 2000; Mack et al., 2001). *WldS* mice have shown resistance to neurodegeneration in multiple models, including Charcot–Marie–Tooth disease 1A (Meyer zu Horste et al., 2011), Parkinson’s disease (Sajadi et al., 2004), and retinal ganglion cell death after optic nerve crush injury (Lorber et al., 2012). These mice have also shown improved motor function, learning, and memory after concussive brain injury relative to wild-type littermates (Fox and Faden, 1998). Although multiple mechanisms have been proposed for how *WldS* mice are protected from axonal degeneration (Wang and Barres, 2012), it has recently been shown *in vitro* that NAD, the metabolite of *WldS/*nicotinamide mononucleotide adenylyltransferase enzymatic activity, is both sufficient and specific to recapitulate the axonal protection seen with the *WldS* mutation, thereby strongly suggesting that NAD is a likely molecular mediator of *WldS* axonal protection (Wang et al., 2015). Accordingly, we investigated whether *WldS* mice might be similarly protected from blast-induced TBI, using measures of both neurodegeneration and behavioral outcome.

## Materials and Methods

### Animals

All animal procedures were performed in accordance with the University of Iowa Carver College of Medicine animal care committee’s regulations. Animals were housed in temperature-controlled conditions, provided food and water *ad libitum*, and maintained on a 12-h light/dark cycle (6 a.m. to 6 p.m.). Heterozygous *WldS* mice (kindly provided by Dr. Karen O’Malley of Washington University, St. Louis, MO) were bred to generate *WldS*-positive mice and wild-type littermates. Genotyping was performed using genotyping primers: forward, CGTTGGCTCTAAGGACAGCAC, and reverse, CTGCAGCCCCCACCCCTT. Mice used were male and 8 weeks of age at the time of injury.

### Blast-mediated TBI

Mice were anesthetized with 1 mg/kg ketamine and 0.1 mg/kg xylazine and placed in an enclosed blast chamber (50 cm long and 33 cm wide) constructed from an air tank partitioned into two sides. One side was pressurized with a 13-cm opening between the partitions and covered with a Mylar membrane. The unpressurized partition contained a restraint 10 cm from the Mylar membrane, into which the mouse was placed. The head was freely moving, whereas a metal tube shielded the body. Compressed air was forced into the pressurized partition until the Mylar membrane burst at 27 kPa. The blast wave impacted the test animal inside a foam-lined restraint to reduce blunt impact trauma of the head against the metal tube. The left side of the head was closest to the origin of the blast wave. Sham-injured animals were anesthetized in the same way and not subjected to the blast.

### Barnes maze

The Barnes maze test was conducted on a gray circular surface 91 cm in diameter, raised to a height of 90 cm, with 20 holes 5 cm in diameter equally spaced around the perimeter (Stoelting Co.). The surface was brightly lit and open to motivate the test animal to learn the location of a dark escape chamber recessed under one of the 20 holes, which was designated randomly. The maze was surrounded by a black circular curtain on which were hung four different and equally spaced visual cues (with different shapes and colors), for orientation to the designated location of the escape chamber. Each animal was subjected to 4 days of training comprising four trials per day. An area extending 4 cm from the escape hole in all directions was used as the target area for measurements (percent time in escape area, percent latency to escape, and nose pokes). A probe trial was conducted on the next day, during which time the escape chamber was removed and measurements were made to confirm the animal’s memory based on spatial cues. Measurements were acquired with Anymaze video tracking software (Stoelting Co.), and analysis was conducted blind to treatment group.

### Foot slip assay

We used standard procedures described by Luong et al. (2011) to measure motor balance coordination. During the training period, mice were trained to cross the 80-cm beam to enter a black box with nesting material three times a day over two consecutive days. On test day, behavior was videotaped during the task, and foot slips were analyzed by an observer blind to condition and treatment group.

### Provocative pattern-evoked electroretinography

Provocative pattern-evoked electroretinography (pPERG) was used to objectively measure the function of retinal ganglion cells by recording the amplitude of the PERG waveform 4 weeks after TBI. Mice were anesthetized with a combination of ketamine (0.03 mg/g, i.p.) and xylazine (0.005 mg/g, i.p.) and placed on a heated recording table to maintain body temperature. They were placed in a 60° head-down position using a custom-made PERG system, and responses were evoked using alternating, reversing, and black-and-white vertical stimuli delivered on a monitor (Jorvec, Miami, FL). A reference needle electrode was placed at the base of the head, and a ground electrode was placed at the base of the tail to complete the circuit. Each animal was placed at the same fixed position in front of the monitor to prevent recording variability caused by animal placement. Mice were positioned in a provocative head-down position for 15 min before initiation of the recording and remained in this position throughout the duration of the recording. Stimuli (18° radius visual angle subtended on full-field pattern, two reversals/s, 372 averaged signals with cutoff filter frequencies of 1–30 Hz, 98% contrast, 80 cd/m^2^ average monitor illumination intensity) were delivered under mesopic conditions without dark adaptation to exclude the possible effect of direct photoreceptor-derived evoked responses. A diffuser placed over the pattern on the monitor also did not elicit a measurable evoked potential, further ensuring that the electrical responses were elicited from retinal ganglion cells. The PERG response was evaluated by measuring the amplitude (peak to trough) of the waveform.

### Immunohistochemistry

Mice were killed by transcardial perfusion with 4% paraformaldehyde at pH 7.4, and dissected brains were immersed in 4% paraformaldehyde overnight at 4°C and cryoprotected in sucrose for 72 h. Brains were then rapidly frozen in isopentane precooled to –70°C with dry ice. All brains were stored in a freezer at –80°C before sectioning. Serial sections (40 µm) were cut coronally through the cerebrum, approximately from bregma 3.20 mm to bregma –5.02 mm, and the brainstem and cerebellum, approximately from bregma –5.52 mm to bregma –6.96 mm (Paxinos and Franklin, 1997). Every section in a series of 12 sections (interval: 480 µm) was collected separately. All sections were stored free-floating in FD sections storage solution (FD Neurotechnologies, Columbia, MD) at –20ºC before further processing. For silver staining, sections were collected in 0.1 m phosphate buffer (pH 7.4) containing 4% paraformaldehyde and fixed for 5 days at 4°C. Sections were then processed for the detection of neurodegeneration with FD NeuroSilver Kit II (FD Neurotechnologies) according to the manufacturer’s instructions. Sections were subsequently mounted on slides, dehydrated in ethanol, cleared in xylene, and coverslipped with Permount (Fisher Scientific, Fair Lawn, NJ). All images were taken with an Aperio ScanScope (Leica Biosystems, Buffalo Grove, IL).

### Immunohistochemistry quantification

Optical densitometry for quantification of immunohistochemical signal was modified from published methodology (Baldock and Poole et al. 1993). Images were captured with an upright microscope (Zeiss Axiolmager.M2) equipped with a monochromatic digital camera (Zeiss AxioCam MRm Rev.3) and processed with Zen imaging software (Zeiss 2012, Blue edition). The microscope light intensity and camera exposure were held constant. The operator outlined areas of interest around specific brain regions and recorded the intensity of light passing through the slide. Degenerating axons allowed less light to pass through the section owing to their uptake of silver stain, so lower light intensity correlated with increased degeneration. The operator performing quantification was blinded to condition and treatment.

### Transmission electron microscopy

Mice were transcardially perfused with Karnovsky’s fixative solution (2% formaldehyde, 2.5% glutaraldehyde, 0.2 m sodium cacodylate buffer, 1 mm CaCl_2_, 2 mm MgCl_2_, and 42.8 mm NaCl, pH 7.4) 2 weeks after either sham or blast injury. Harvested brains were incubated in Karnovsky’s fixative solution overnight at 4°C. Whole brains were cut in the horizontal plane (100 µm) using a vibratome (Leica 1500). Sections that contained the hippocampus were selected, washed with 0.1 m sodium cacodylate buffer, and postfixed with 1% osmium fixative for 1 h. Sections were then dehydrated in a series of ethanol (50%, 75%, 95%, and 100%) followed by embedding in EPON resin overnight at 65°C. For transmission electron microscopy, ultrathin sections (60 nm) adjacent to semithin sections were cut with an ultramicrotome, loaded onto a Formvar 200-mesh Ni grid, and counterstained with uranyl acetate and lead citrate. Specimens were examined using a JEOL JEM 1230 electron microscope with a Gatan UltraScan 1000 2k x 2k charge-coupled device camera.

### Statistical analysis

All data was compiled and analyzed using Graphpad Prism. Significance was performed with ANOVA and Tukey post hoc analysis.

## Results

### *WldS* mice are protected from learning and memory deficits after blast-mediated TBI

To evaluate the susceptibility of *WldS* mice to blast-mediated TBI, we used a model of blast injury in which a blast wave is intiated by rupture of a mylar membrane to expose anesthetized mice in an enclosed overpressure chamber composed of an air tank partitioned into two sides (Mohan et al., 2013; Yin et al., 2014)). A sealed mylar membrane covers a port between the two parts of the tank, and pressure is increased in the side without the mouse until the membrane ruptures at ∼27 kPa. This rupture generates a blast wave that travels through the mouse’s untethered head located in a padded holder, while the body is shielded by a metal tube. The intensity of the blast wave is 149.8 ± 2.09 kPa, and total duration of the pressue is ∼10–15 ms, composed of both blast wave and wind gust (Mohan et al., 2013; Yin et al., 2014).

**Figure 1.**
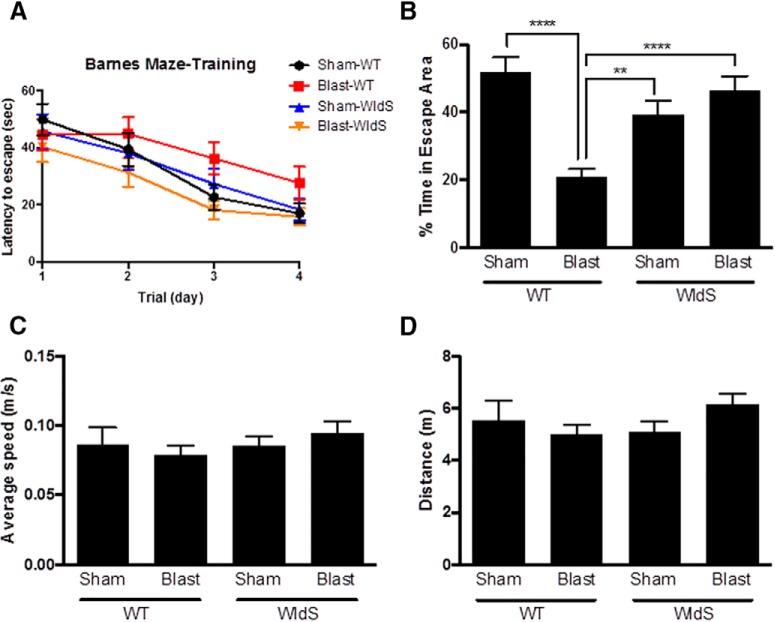
*WldS* mice are protected from memory deficits after blast-mediated TBI. ***A***, Latency to escape progressively decreases over the 4-day training period in all groups. ***B***, Blast-mediated TBI wild-type (WT) mice spend less than half the time as sham-injury WTs in the escape area (5-cm radius around the escape hole) during the probe test of memory. Blast-mediated TBI *WldS* mice spend a amount of time in the escape area comparable to that of sham-injury WT and sham-injury *WldS* mice. ***C***, The average locomotion speed during the probe trial was similar in all groups. ***D***, The total distance traveled during the probe trial was similar in all groups. Each group consisted of 25 male congenic C57/Bl6 mice, aged 12–14 weeks. Data were collected and scored in an automated manner blind to treatment group. Data are represented as mean ± SEM. Significance was determined by two-way ANOVA with Bonferroni post hoc analysis. *p*-values labeled as **<0.01 and ****<0.0001 compared with blast-injured WT animals.

The Barnes maze was used to evaluate hippocampal-dependent spatial learning in *WldS* mice after blast-induced TBI, with 25 animals per group. This task consists of a round table with equally spaced holes at its perimeter, one of which contains an escape cup. Mice are motivated to learn the location of the hole that houses the escape cup, so that they can enter the hole and hide in the cup to avoid exposure on the table. Testing was initiated 7 days after blast injury, beginning with 4 days of training in which mice were allowed to find and enter the escape hole and then rest in the protective cup. All mice, regardless of genotype or injury group, learned how to locate the platform more quickly over the course of the 4-day training period (Fig. 1*A*), indicating equal ability to learn in all four groups. On day 5, the probe test was conducted, in which the escape cup was removed and the ability of the mouse to remember the location of the cup was then assessed by measuring the amount of time the mouse spent in the area surrounding where the cup had been previously located. During the probe test, sham-injured wild-type animals spent ∼50% of their time in the escape area, defined as a 5-cm radius surrounding the escape hole (Fig. 1*B*). This indicates normal memory. There was no significant difference in performance in the probe test between sham-injured *WldS* mice and sham-injured wild-type littermate mice. In contrast, blast-injured wild-type mice spent only ∼20% of their time in the escape area (Fig. 1*B*; *p* < 0.0001 relative to sham-injury wild-type), indicating the expected degree of impaired memory after injury (Yin et al., 2014). No differences in this measure were seen between sham-injury and blast-injury *WldS* groups, in relation to each other or sham-injury wild-type mice. Importantly, both sham-injury and blast-injury *WldS* mice showed significantly greater time in the escape area than blast-injury wild-type mice, with ∼40% time in escape area for sham-injury *WldS* (*p* < 0.01 relative to sham-injury wild-type) and ∼43% time in escape area for blast-injury *WldS* (*p* < 0.0001 relative to sham-injury wild-type; Fig. 1*B*). This indicates that whereas the *WldS* mutation does not improve the animal’s memory under normal conditions, it does effectively block impairment in memory that is normally observed after blast injury. Importantly, none of the four groups differed in ability to physically participate in the task, as determined by comparable levels of average speed of locomotion and total distance traveled during the probe test (Fig 1*C*, *D*).

### *WldS* mice are protected from motor coordination deficits after blast-mediated TBI

**Figure 2.**
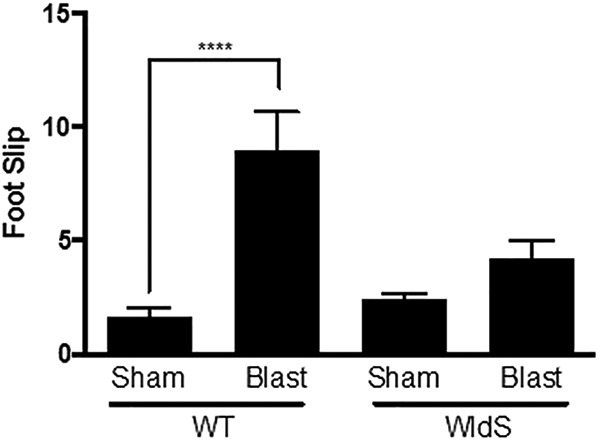
*WldS* mice are protected from motor coordination deficits after blast-mediated TBI. Blast-injured wild-type (WT) mice showed an increased number of foot slips relative to sham-injury WT mice 28 days after blast-mediated TBI. Blast-injured *WldS* mice show a similar number of foot slips as sham-injury WT and sham-injury *WldS* mice. Each group consisted of 25 male congenic C57/Bl6 mice, aged 12–14 weeks. Data was manually collected and scored blind to treatment group. Data are represented as mean ± SEM. Significance was determined by two-way ANOVA with Bonferroni post hoc analysis. Significance was determined by two-way ANOVA with Bonferroni post hoc analysis. *p*-values labeled as ****<0.0001 compared to sham-injured WT animals.

To assay balance and coordination, which are compromised after blast-mediated TBI in wild-type mice (Yin et al., 2014), we used the standard balance beam task (Luong et al., 2011). Mice were trained to cross an 80-cm-long balance beam over 2 days before the day of injury, and tested again 28 days after injury. Videorecording of all mice traversing the beam was analyzed for the number of foot slips by observers blind to genotype or injury group. Blast-injured wild-type mice displayed four times as many foot slips as sham-injured wild-type mice (*p* < 0.0001 relative to sham-injury wild-type), whereas sham- and blast-injury *WldS* mice showed no difference from sham-injury wild-type mice (Fig. 2). Thus, *WldS* mice are protected from the motor coordination deficits that are normally observed after blast-mediated TBI in mice.

### *WldS* mice are protected from axonal degeneration after blast-mediated TBI

**Figure 3.**
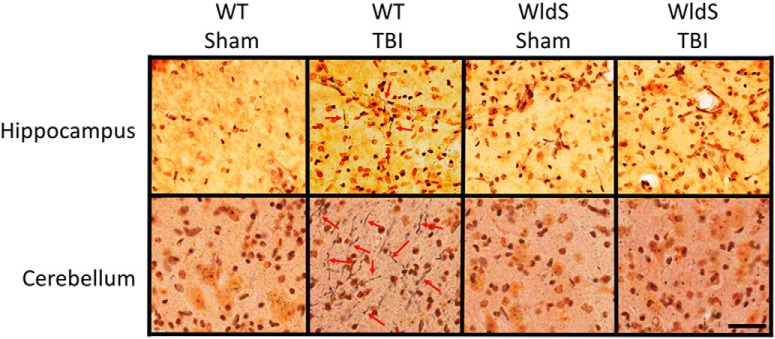
*WldS* hippocampus and cerebellum are protected from axonal degeneration after blast-mediated TBI. High-power representative pictures with 40× objective from hippocampal CA1 stratum radiatum and cerebellum show prominent silver staining of degenerating axons (red arrows) in blast-injured wild-type mice 12 days after injury, with little to no axonal degeneration in *WldS* mice after the same injury. Images shown are representative of typical images from five animals in each group. Scale bar = 2.5 mm.

**Figure 4.**
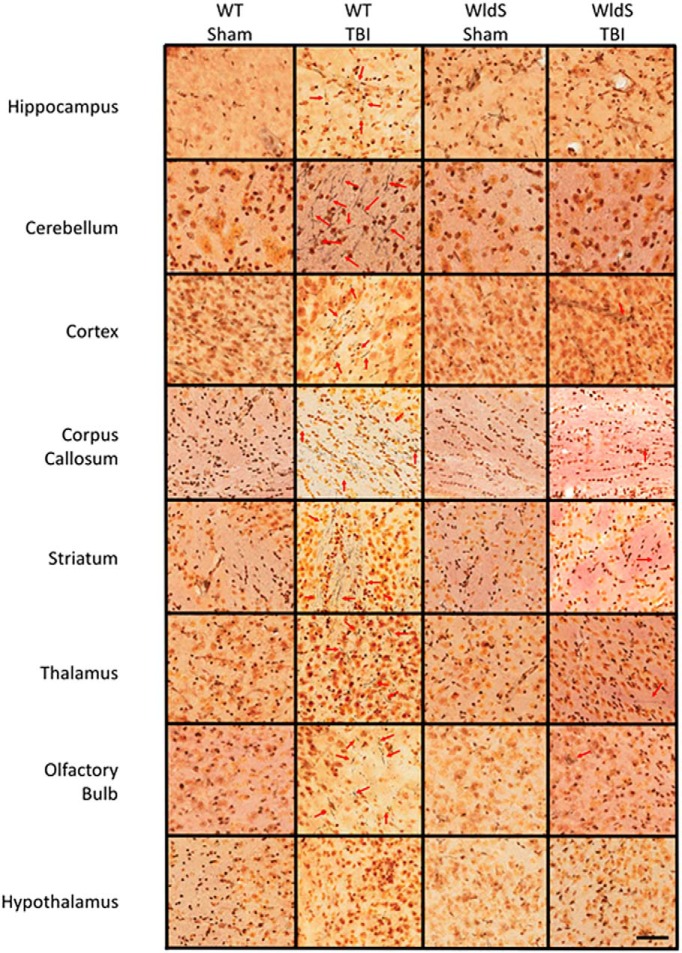
*WldS* mice are broadly protected throughout the brain from axonal degeneration after blast-mediated TBI. As in hippocampus and cerebellum, protection was also noted in *WldS* cortex, corpus callosum, olfactory bulb, striatum, and thalamus. Noticeably, the hypothalamus is resistant to axonal degeneration in wild-type mice after blast-mediated TBI. Images shown are representative of typical images from five animals in each group. Scale bar = 2.5 mm.

**Figure 5.**
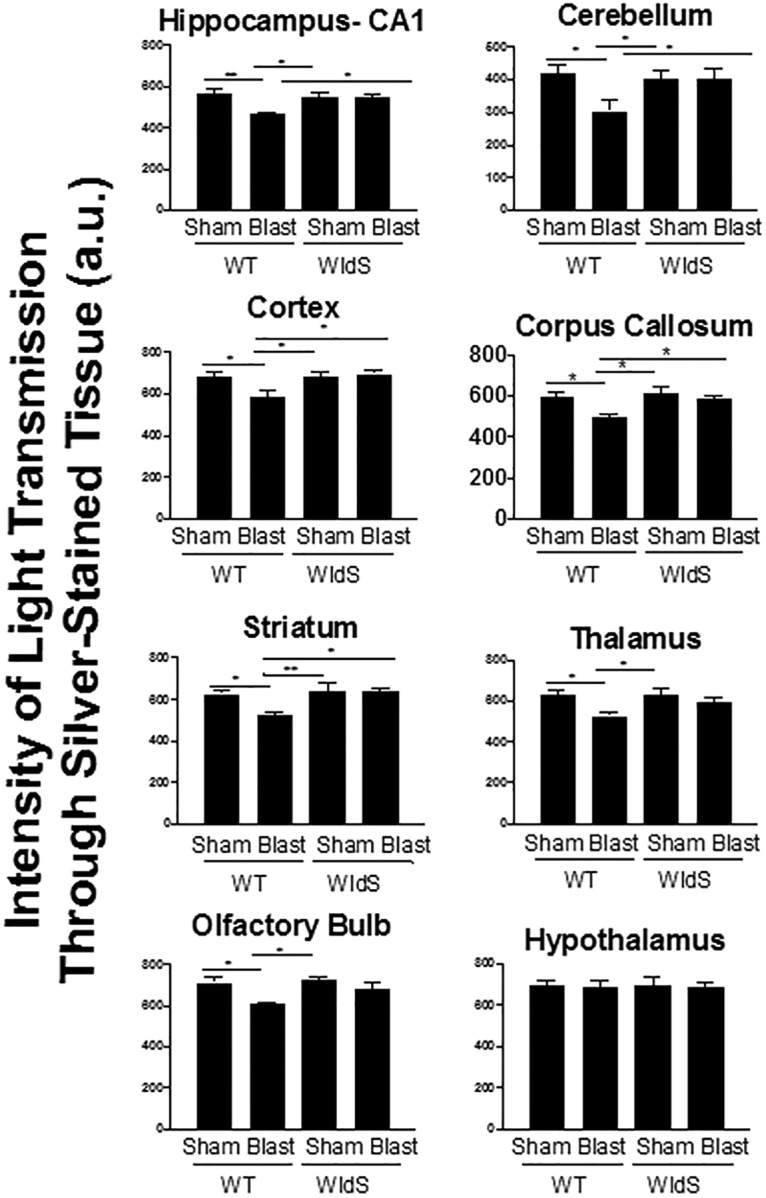
Optical densitometry of light transmitted through silver-stained brain regions from all animals in each group was used to quantify the protective effect. Signal was quantified for 18 sections for each of the five animals, spaced 480 mm apart. Here, a greater value indicates that more light was able to pass unimpeded through the section by virtue of less silver staining, which reflects less axonal degeneration. Data are represented as mean ± SEM. *p*-value *<0.05 and **<0.01 determined by two-way ANOVA with Bonferroni post hoc analysis compared with blast-injured WT animals.

Histologic examination of brain tissue for evidence of axonal degeneration was conducted via silver staining 12 days after injury, as previously established (Yin et al., 2014). In wild-type mice, blast injury was associated with prominent silver staining of degenerating axons in CA1 stratum radiatum of the hippocampus, cerebellum, cortex, corpus callosum, olfactory bulb, striatum, and thalamus, with no injury in the hypothalamus (Figs. 3 and 4), consistent with previous observations in this TBI model (Yin et al., 2014). Use of an established technique of automated optical densitometry to quantify the magnitude of silver staining, in which decreased signal indicates greater impedence of light through the tissue owing to silver staining of degenerating axons (Yin et al., 2014), showed that in all cases the extent of axonal degeneration in wild-type mice after TBI was statistically significant compared with the sham group (Fig. 5). There were no signficant differences between sham-injured *WldS* mice and sham-injured wild-type littermate mice. In addition, no significant differences were noted between sham- and blast-injury *WldS* mice, and both of these groups also showed statistically greater signal than blast-injury wild-type mice in CA1 stratum radiatum of the hippocampus, cerebellum, cortex, corpus callosum, and striatum (Fig. 5).

**Figure 6.**
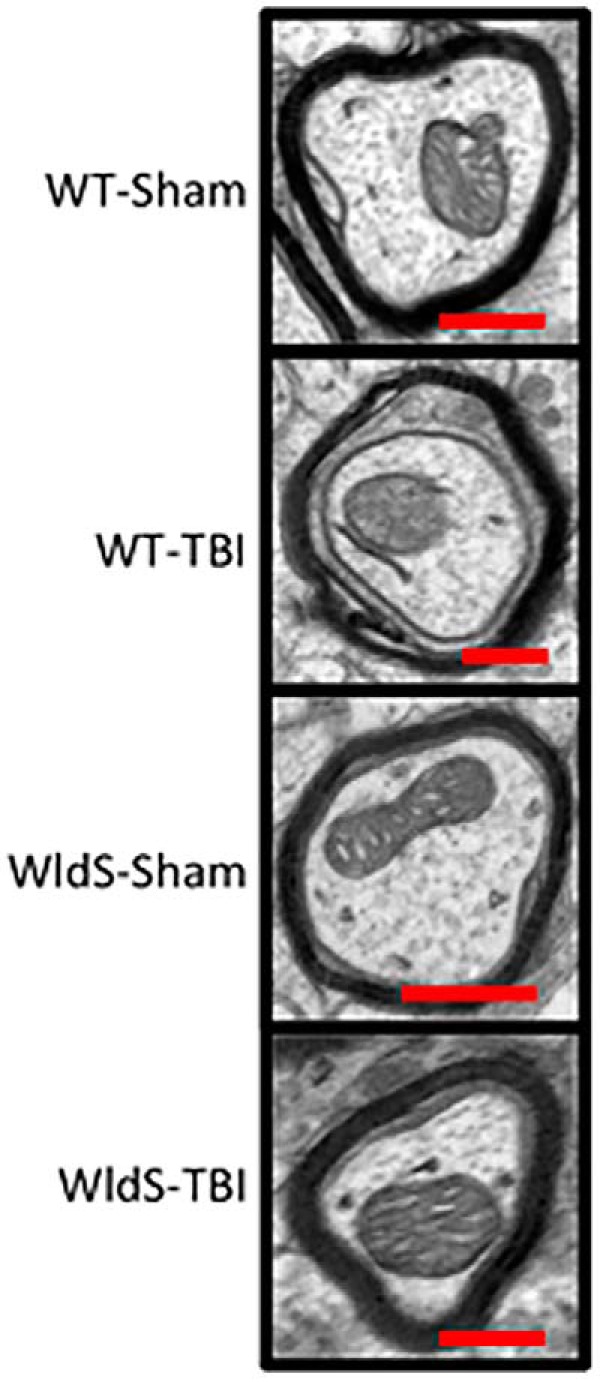
Transmission electron microscopy 12 days after injury shows normal myelin and axonal mitochondrial structures in the CA1 stratum radiatum of sham-injury wild-type mice, with no differences seen in sham- and blast-injury *WldS* mice. Blast-injured wild-type mice show degeneration of the myelin sheath along, as well as abnormal outer membrane and internal cristae structures within neuronal mitochondria.

Protective efficacy of the *WldS* mutation was confirmed by transmission electron microscopy of brain tissue 12 days after injury. This showed normal myelin and axonal mitochondrial structures in the CA1 stratum radiatum of sham-injury wild-type mice, as well as in sham- and blast-injury *WldS* mice (Fig. 6). Blast-injured wild-type mice, however, showed degeneration of the myelin sheath, as well as abnormal outer membrane and internal cristae structures within neuronal mitochondria.

### *WldS* mice are protected from damage to the visual system after blast-mediated TBI

**Figure 7.**
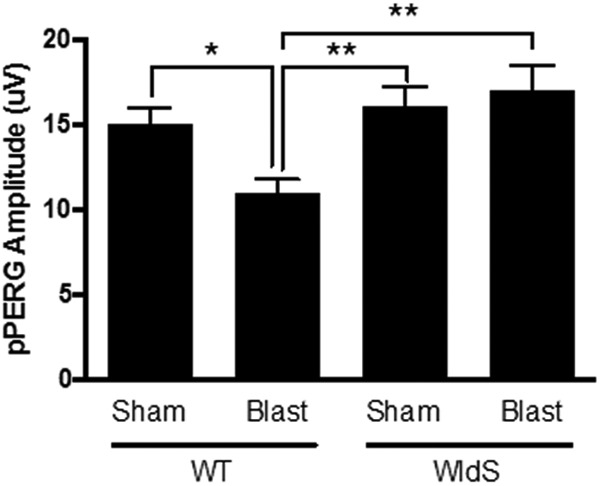
*WldS* mice are protected from pPERG deficits after blast-mediated TBI. pPERG serves as an early indicator of future chronic damage to the visual system, including retinal cell death. Wild-type (WT) mice exhibit ∼25% decrease in pPERG 4 weeks after blast injury, whereas both sham- and blast-injury *WldS* mice exhibit pPERG levels equivalent to sham-injury WT mice. Each group consisted of 25 male congenic C57/Bl6 mice, aged 12–14 weeks. Data were collected and scored in an automated manner blind to identification of the group and are represented as mean ± SEM. *p*-value *<0.05 and **<0.01 determined by-two way ANOVA with Bonferroni post hoc analysis, compared to blast-injured WT animals.

In patients, TBI frequently leads to chronic visual dysfunction, including light sensitivity, ocular motility dysfunction, optic neuropathy, retinopathy, and visual field loss (Cockerham et al., 2009, 2011; Lemke et al., 2013). A measure of retinal ganglion cell and optic nerve damage is the pattern electroretinogram (PERG), a painless and noninvasive objective diagnostic measure of retinal function that requires no verbal communication between patient and clinicians. This latter feature is important, given the acute variance in mental functioning of patients after TBI. PERG is applicable to both human patients and mice, and measures stimulus-evoked electrical activity of retinal cells in response to contrast modulation of patterned visual stimuli, such as a checkerboard, at constant luminance (Porciatti, 2007). Because deficits in PERG typically do not arise until visual damage has been sustained, this procedure has been modified to the provocative PERG (pPERG), in which mice or people are tilted with their head down to increase intraocular pressure. This amplifies retinal sensitivity to damage and has recently been shown to provide an early, sensitive, and noninvasive indicator of future chronic visual dysfunction after TBI, including later development of retinal cell death (Dutca et al., 2014). When wild-type mice were exposed to blast injury, they exhibited significantly decreased pPERG amplitude 4 weeks later, relative to sham injury wild-type animals (*p* < 0.05; Fig. 7). Both sham- and blast-injury *WldS* animals, however, showed pPERG amplitude preserved to the wild-type sham-injury level (*p* < 0.01 relative to blast-injury wild-type animals; Fig. 7), indicating that the *WldS* mutation protects the visual system after TBI.

## Discussion

An estimated 5 million people in the US currently experience long-term motor and cognitive disability related to TBI, at an annual cost exceeding $70 billion (Fleminger and Ponsford, 2005). Although axonal degeneration is a major aspect of many forms of neurodegeneration (Warner et al., 2010; Wang et al., 2012; Johnson et al., 2013; Smith et al., 2013), its contribution to the pathological and neurobehavioral deficits of blast-mediated TBI, the most common form of TBI sustained by soldiers in Iraq and Afghanistan, has not previously been investigated. Here, we show for the first time that early axonal degeneration is a critical driver of the development of neurologic deficits after blast-mediated TBI.

We addressed this issue by using *WldS* mice, which are are resistant to Wallerian degeneration, an active process of axon-autonomous self-destruction linked to neurodegeneration in both injury and disease (Conforti et al., 2014). These mice have been previously shown to be protected from Wallerian degeneration of axons after injury in different regions, including spinal cord (Fujiki et al., 1996; Zhang et al., 1996) and dentate gyrus (Schauwecker and Steward, 1997). Here, we report that these mice are broadly protected from axonal degeneration throughout the brain after blast injury. Importantly, this protection is also associated with complete preservation of normal cognitive, motor, and visual function after blast exposure. These preclinical studies were highly powered and rigorously executed, with data acquisition and analysis conducted blind to genotype and injury group. Given the current lack of efficacious treatment for patients with any form of TBI (Smith et al., 2013), including blast-mediated, these findings are highly clinically relevant. It is not known, however, whether the beneficial effect of *WldS* would also be effective post-injury, and future experiments will be needed to address this question. In addition, future work focused on specifically delineating the role of *WldS* in the distinct hippocampal, cerebellar, and visual neuronal circuitry underlying these disparate behavioral effects could add further insight into unique pathological processes involved in each domain after injury.

Although our results are highly suggestive that early thereapeutic intervention aimed at mitigating axonal degeneration is likely to benefit patients suffering from blast-mediated TBI, they do not provide definitive evidence that the functional improvement in *WldS* mice after blast-mediated TBI is exclusively the result of axonal protection. However, a complementary body of literature supports this notion. For example, it has recently been shown that genetic ablation of the Toll receptor adaptor *sarm1* (sterile α/Armadillo/Toll-Interleukin receptor homology domain protein) gene, which is a key mediator of the active process of Wallerian degeneration, protects mice from multiple injury phenotypes after closed-head mild TBI (Henninger et al., 2016). These findings with a genetic loss-of-function model in TBI nicely complement our current findings with the gain-of-function *WldS* model.

In addition, recent pharmacologic agents shown to enhance flux of the NAD salvage pathway in normal mammalian cells have also demonstrated axonal protection associated with similar behavioral protection when administered after blast-mediated TBI (Yin et al., 2014), as well as behavioral and histological protection in concussive TBI models (Blaya et al., 2014). Furthermore, other avenues of augmenting neuronal NAD levels, such as administration of nicotinamide (Hoane et al., 2006, 2008; Goffus et al., 2010), poly(ADP-ribose) polymerase inhibition (Clark et al., 2007; Stoica et al., 2014), or intranasal delivery of NAD (Won et al., 2012) also show protective efficacy in multiple histologic and behavioral outcome measures after concussive TBI.

Thus, taken together, our findings support the notion that Wallerian degeneration is an important underlying pathological feature of blast-mediated TBI and its behavioral consequences. This illustrates the translational potential of NAD-augmenting therapies known to promote axonal survival, or future alternative approaches for promoting axonal survival, as a fruitful avenue for clinical treatment of patients suffering from the effects of blast-mediated TBI.

## Synthesis

The decision was a result of the Reviewing Editor Michael Sofroniew and the peer reviewers coming together and discussing their recommendations until a consensus was reached. A fact-based synthesis statement explaining their decision and outlining what is needed to prepare a revision is listed below. The following reviewers agreed to reveal their identity: Rajiv Ratan, John Greer

The paper is now acceptable.
